# Supplementary material for: Development of highly sensitive and low-cost DNA agarose gel electrophoresis detection systems, and evaluation of non-mutagenic and loading dye-type DNA-staining reagents
Source: PLoS One. 2019 Sep 9;14(9):e0222209. doi: 10.1371/journal.pone.0222209 (PMC6733488; doi:10.1371/journal.pone.0222209)
Supplement: S1 Fig — Profiles of the detection limit under each condition were analyzed by “Plot Profile” of Image-J. Each excitation system is represented with boxed letters, and each DNA-staining reagent is represented by underlined letters. Blue arrows indicate detectable DNA bands. (a) Fig 1A left, lane 5; (b) Fig 1A right, lane 4; (c) Fig 1B left, lane 3; (d) Fig 1C left, lane 5; (e) Fig 1V middle, lane 3; (f) Fig 1V right, lane 5; (g) Fig 3A left, lane 1; (h) Fig 3A right, lane 1; (i) Fig 3B left, lane 1; (j) Fig 3B right, lane 1. (PPTX) [file pone.0222209.s001.pptx]

## Slide 1
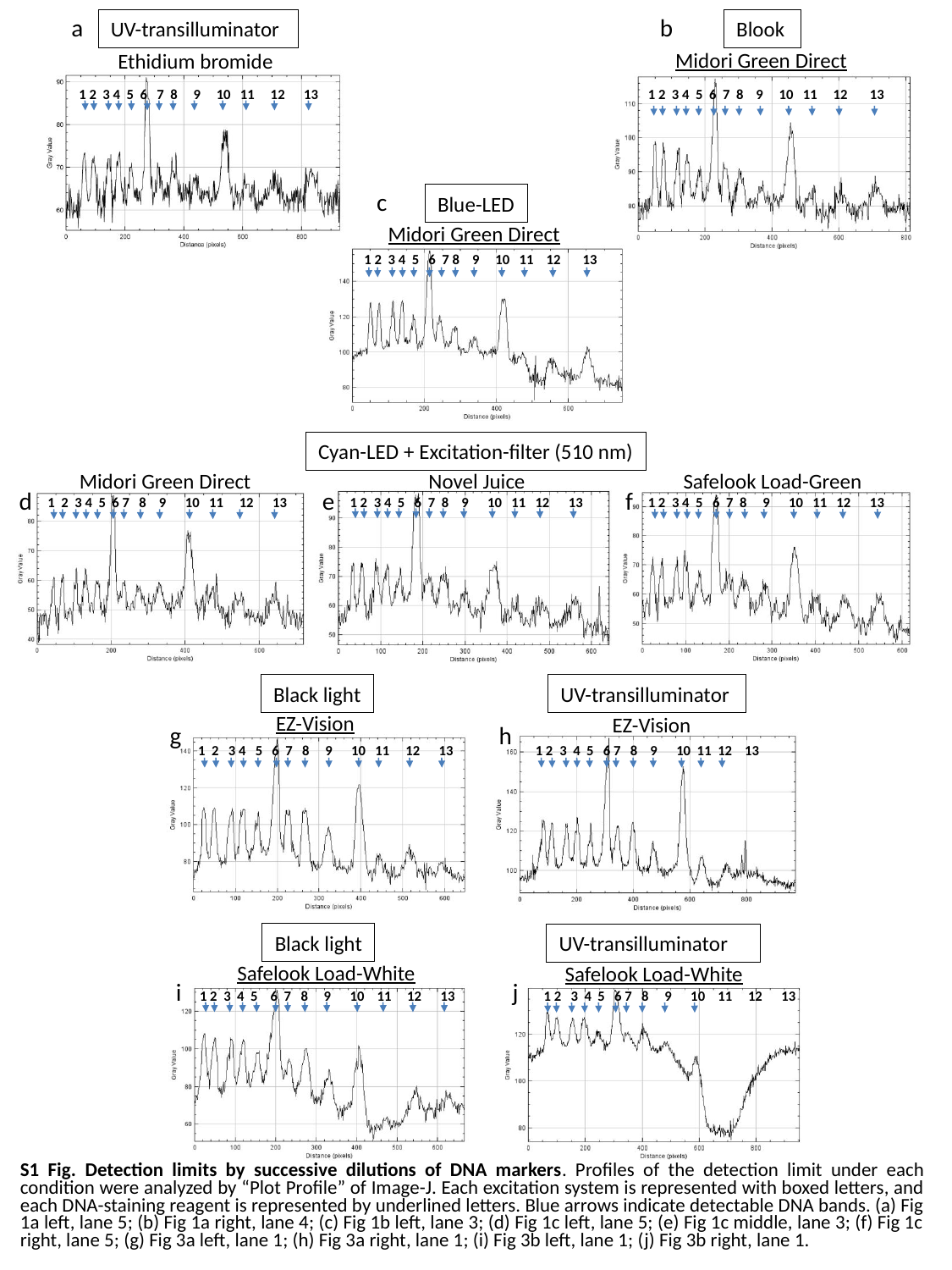

a
b
Blook
Midori Green Direct
UV-transilluminator
Ethidium bromide
1 2 3 4 5 6 7 8 9 10 11 12 13
1 2 3 4 5 6 7 8 9 10 11 12 13
c
Blue-LED
Midori Green Direct
1 2 3 4 5 6 7 8 9 10 11 12 13
Cyan-LED + Excitation-filter (510 nm)
Midori Green Direct
Novel Juice
Safelook Load-Green
d
e
f
1 2 3 4 5 6 7 8 9 10 11 12 13
1 2 3 4 5 6 7 8 9 10 11 12 13
1 2 3 4 5 6 7 8 9 10 11 12 13
Black light
EZ-Vision
UV-transilluminator
EZ-Vision
g
h
1 2 3 4 5 6 7 8 9 10 11 12 13
1 2 3 4 5 6 7 8 9 10 11 12 13
Black light
Safelook Load-White
UV-transilluminator
Safelook Load-White
i
j
1 2 3 4 5 6 7 8 9 10 11 12 13
1 2 3 4 5 6 7 8 9 10 11 12 13
S1 Fig. Detection limits by successive dilutions of DNA markers. Profiles of the detection limit under each condition were analyzed by “Plot Profile” of Image-J. Each excitation system is represented with boxed letters, and each DNA-staining reagent is represented by underlined letters. Blue arrows indicate detectable DNA bands. (a) Fig 1a left, lane 5; (b) Fig 1a right, lane 4; (c) Fig 1b left, lane 3; (d) Fig 1c left, lane 5; (e) Fig 1c middle, lane 3; (f) Fig 1c right, lane 5; (g) Fig 3a left, lane 1; (h) Fig 3a right, lane 1; (i) Fig 3b left, lane 1; (j) Fig 3b right, lane 1.
