# Supplementary material for: Development of highly sensitive and low-cost DNA agarose gel electrophoresis detection systems, and evaluation of non-mutagenic and loading dye-type DNA-staining reagents
Source: PLoS One. 2019 Sep 9;14(9):e0222209. doi: 10.1371/journal.pone.0222209 (PMC6733488; doi:10.1371/journal.pone.0222209)
Supplement: S2 Fig — (a) Blue-LED (Compact LED viewer, Sanplatec); (b) Left, cyan-LED (hlk-12led-490nm-495nm, Holkin), right, cyan-LED with excitation filter (SV0510; Asahi Spectra); (c) left, compact black light blue lamp 27 W (360 nm, FPL27BLB; Sankyo Denki), right, black light to be mounted on a fluorescent lamp stand. (PPTX) [file pone.0222209.s002.pptx]

## Slide 1
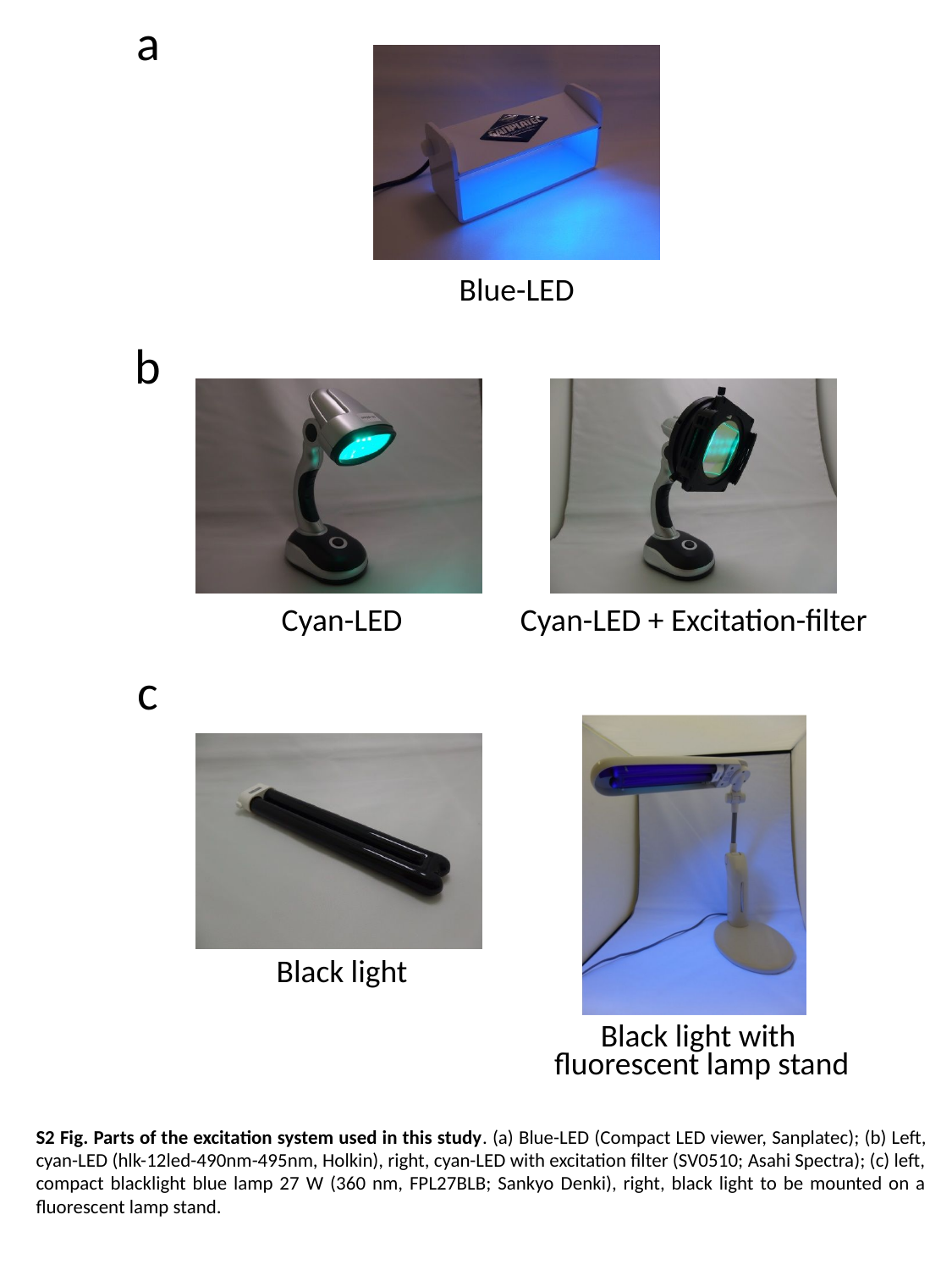

a
Blue-LED
b
Cyan-LED
Cyan-LED + Excitation-filter
c
Black light
Black light with
fluorescent lamp stand
S2 Fig. Parts of the excitation system used in this study. (a) Blue-LED (Compact LED viewer, Sanplatec); (b) Left, cyan-LED (hlk-12led-490nm-495nm, Holkin), right, cyan-LED with excitation filter (SV0510; Asahi Spectra); (c) left, compact blacklight blue lamp 27 W (360 nm, FPL27BLB; Sankyo Denki), right, black light to be mounted on a fluorescent lamp stand.
