# Supplementary material for: Development of highly sensitive and low-cost DNA agarose gel electrophoresis detection systems, and evaluation of non-mutagenic and loading dye-type DNA-staining reagents
Source: PLoS One. 2019 Sep 9;14(9):e0222209. doi: 10.1371/journal.pone.0222209 (PMC6733488; doi:10.1371/journal.pone.0222209)
Supplement: S3 Fig — Gel images were recorded with a digital camera PowerShot G12 (Canon) in a dark place shaded by blackout curtain. (a) Blue-LED system; (b) Cyan-LED + Excitation filter system; (c) Black light system. (PPTX) [file pone.0222209.s003.pptx]

## Slide 1
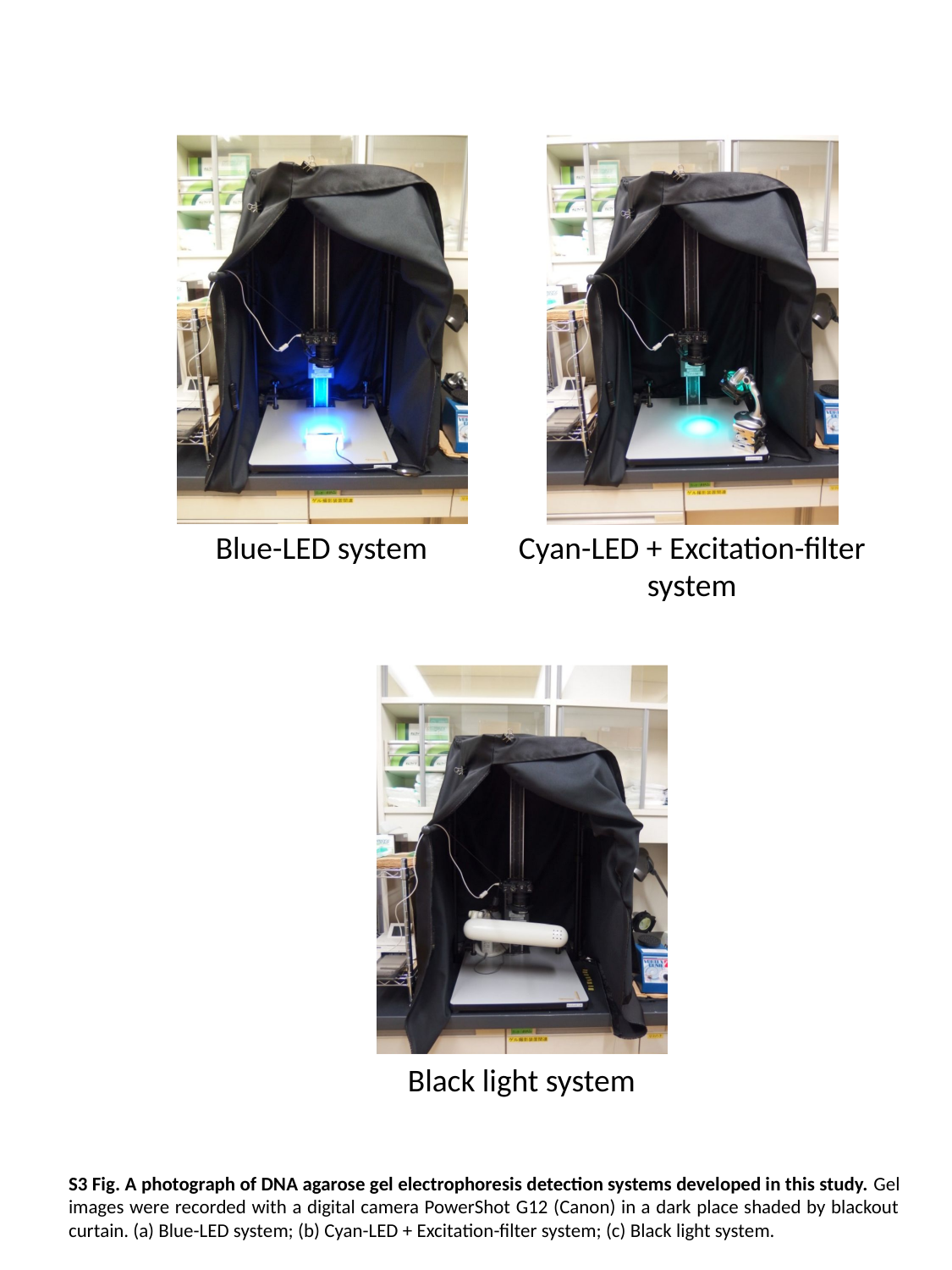

Blue-LED system
Cyan-LED + Excitation-filter
system
Black light system
S3 Fig. A photograph of DNA agarose gel electrophoresis detection systems developed in this study. Gel images were recorded with a digital camera PowerShot G12 (Canon) in a dark place shaded by blackout curtain. (a) Blue-LED system; (b) Cyan-LED + Excitation-filter system; (c) Black light system.
