# Supplementary material for: Development of highly sensitive and low-cost DNA agarose gel electrophoresis detection systems, and evaluation of non-mutagenic and loading dye-type DNA-staining reagents
Source: PLoS One. 2019 Sep 9;14(9):e0222209. doi: 10.1371/journal.pone.0222209 (PMC6733488; doi:10.1371/journal.pone.0222209)
Supplement: S4 Fig — (a) Excitation by blue-LED light (470 nm); (b) Excitation by cyan-LED light (490–495 nm); (c) Excitation by combination of cyan LED (490–495 nm) and a shortpass filter (510 nm). SC-52, SC-54, and SC-56 filters were evaluated as longpass emission-filters. DNA ladder markers were loaded by successive dilution. Lane 1, standard volume (5 μL (500 ng), 1 volume); lane 2, 1/2 volume; lane 3, 1/3 volume; lane 4, 1/6 volume; lane 5, 1/10 volume; lane 6, 1/15 volume; lane 7, 1/20 volume; lane 8, 1/30 volume. (PPTX) [file pone.0222209.s004.pptx]

## Slide 1
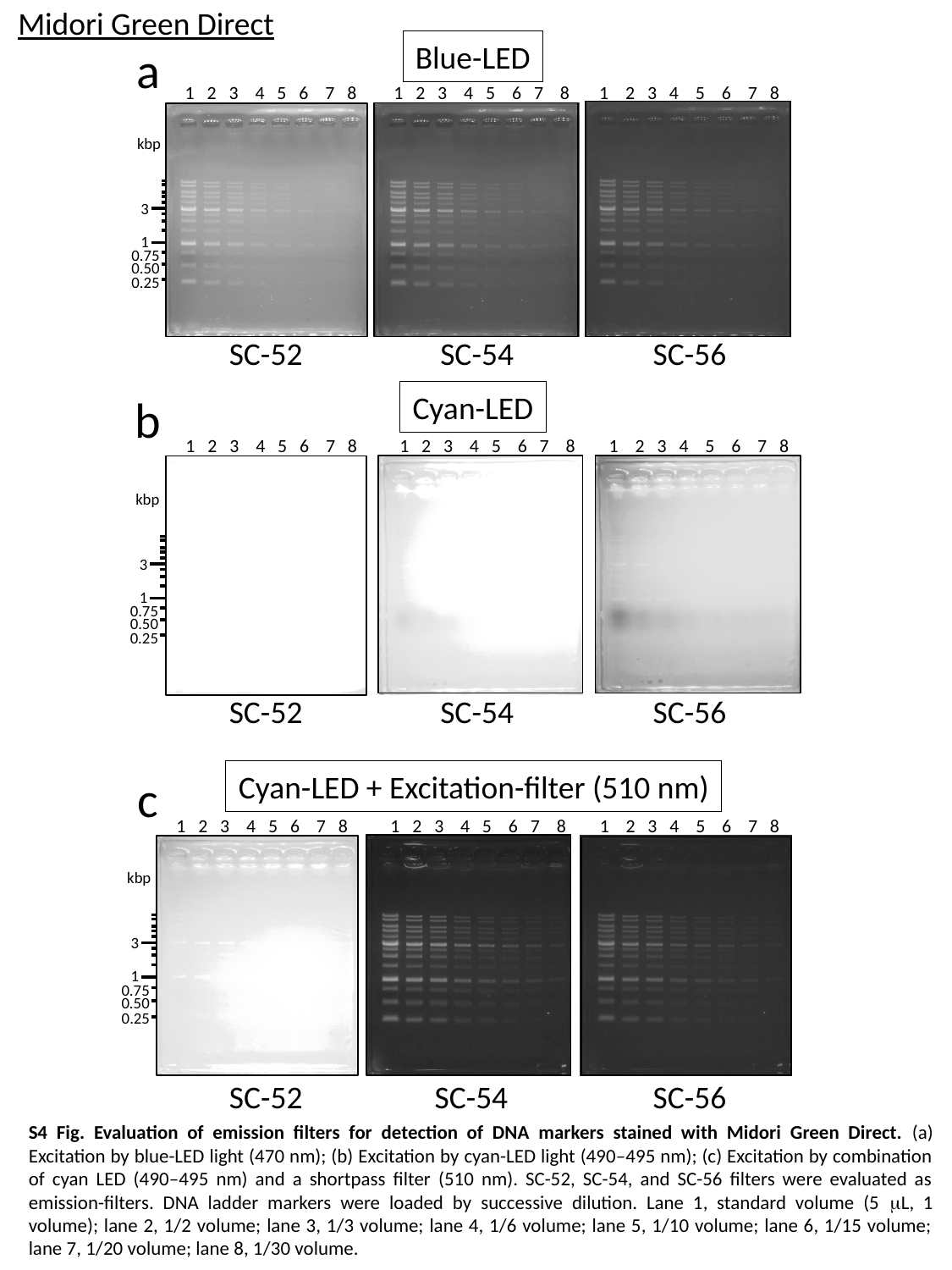

Midori Green Direct
Blue-LED
a
1 2 3 4 5 6 7 8
1 2 3 4 5 6 7 8
1 2 3 4 5 6 7 8
kbp
3
1
0.75
0.50
0.25
SC-52
SC-54
SC-56
Cyan-LED
b
1 2 3 4 5 6 7 8
1 2 3 4 5 6 7 8
1 2 3 4 5 6 7 8
kbp
3
1
0.75
0.50
0.25
SC-52
SC-54
SC-56
Cyan-LED + Excitation-filter (510 nm)
c
1 2 3 4 5 6 7 8
1 2 3 4 5 6 7 8
1 2 3 4 5 6 7 8
kbp
3
1
0.75
0.50
0.25
SC-52
SC-54
SC-56
S4 Fig. Evaluation of emission filters for detection of DNA markers stained with Midori Green Direct. (a) Excitation by blue-LED light (470 nm); (b) Excitation by cyan-LED light (490–495 nm); (c) Excitation by combination of cyan LED (490–495 nm) and a shortpass filter (510 nm). SC-52, SC-54, and SC-56 filters were evaluated as emission-filters. DNA ladder markers were loaded by successive dilution. Lane 1, standard volume (5 mL, 1 volume); lane 2, 1/2 volume; lane 3, 1/3 volume; lane 4, 1/6 volume; lane 5, 1/10 volume; lane 6, 1/15 volume; lane 7, 1/20 volume; lane 8, 1/30 volume.
