# Supplementary material for: Development of highly sensitive and low-cost DNA agarose gel electrophoresis detection systems, and evaluation of non-mutagenic and loading dye-type DNA-staining reagents
Source: PLoS One. 2019 Sep 9;14(9):e0222209. doi: 10.1371/journal.pone.0222209 (PMC6733488; doi:10.1371/journal.pone.0222209)
Supplement: S5 Fig — (a) Detection of DNA markers stained with EZ-Vision; (b) Detection of DNA markers stained with Safelook Load-White. Black light (~360 nm) was used to excite EZ-Vision and Safelook Load-White. SC-42, SC-46, and SC-48 filters were evaluated as longpass emission filters. DNA ladder markers were loaded by successive dilution. Lane 1, standard volume (5 μL (500 ng), 1 volume); lane 2, 1/2 volume; lane 3, 1/3 volume; lane 4, 1/6 volume; lane 5, 1/10 volume; lane 6, 1/15 volume; lane 7, 1/20 volume; lane 8, 1/30 volume. (PPTX) [file pone.0222209.s005.pptx]

## Slide 1
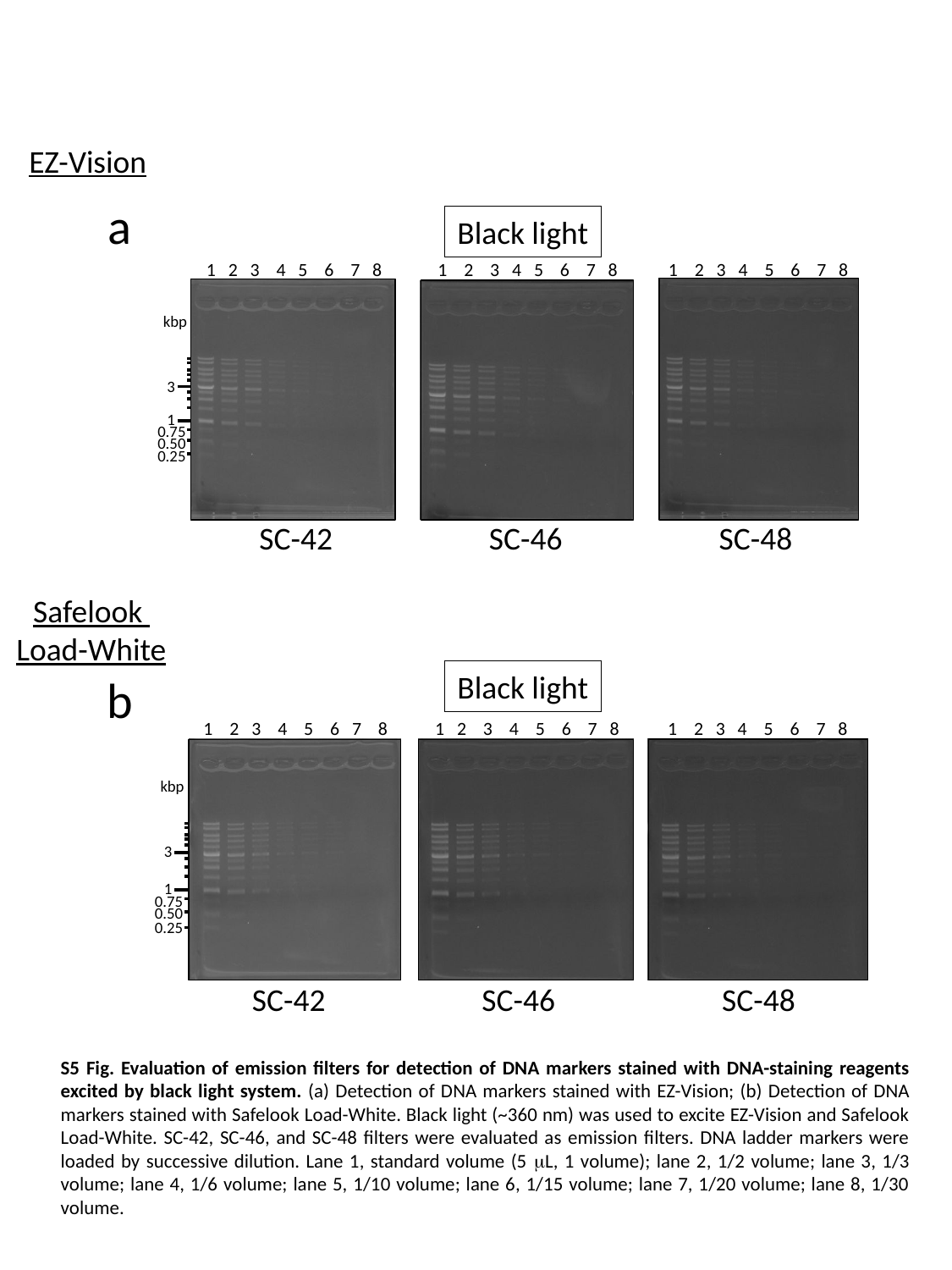

EZ-Vision
a
Black light
1 2 3 4 5 6 7 8
1 2 3 4 5 6 7 8
1 2 3 4 5 6 7 8
kbp
3
1
0.75
0.50
0.25
SC-42
SC-46
SC-48
Safelook
Load-White
Black light
b
1 2 3 4 5 6 7 8
1 2 3 4 5 6 7 8
1 2 3 4 5 6 7 8
kbp
3
1
0.75
0.50
0.25
SC-42
SC-46
SC-48
S5 Fig. Evaluation of emission filters for detection of DNA markers stained with DNA-staining reagents excited by black light system. (a) Detection of DNA markers stained with EZ-Vision; (b) Detection of DNA markers stained with Safelook Load-White. Black light (~360 nm) was used to excite EZ-Vision and Safelook Load-White. SC-42, SC-46, and SC-48 filters were evaluated as emission filters. DNA ladder markers were loaded by successive dilution. Lane 1, standard volume (5 mL, 1 volume); lane 2, 1/2 volume; lane 3, 1/3 volume; lane 4, 1/6 volume; lane 5, 1/10 volume; lane 6, 1/15 volume; lane 7, 1/20 volume; lane 8, 1/30 volume.
