# Supplementary material for: Development of highly sensitive and low-cost DNA agarose gel electrophoresis detection systems, and evaluation of non-mutagenic and loading dye-type DNA-staining reagents
Source: PLoS One. 2019 Sep 9;14(9):e0222209. doi: 10.1371/journal.pone.0222209 (PMC6733488; doi:10.1371/journal.pone.0222209)
Supplement: S6 Fig — (a) Orange spectacles (UVP, UVC-310) can be used as an alternative to SC-54, which is an orange filter. (b) Yellow spectacles (TRUSCO, TSG-814Y) can be used as an alternative to SC-46, which is a yellow filter. (PPTX) [file pone.0222209.s006.pptx]

## Slide 1
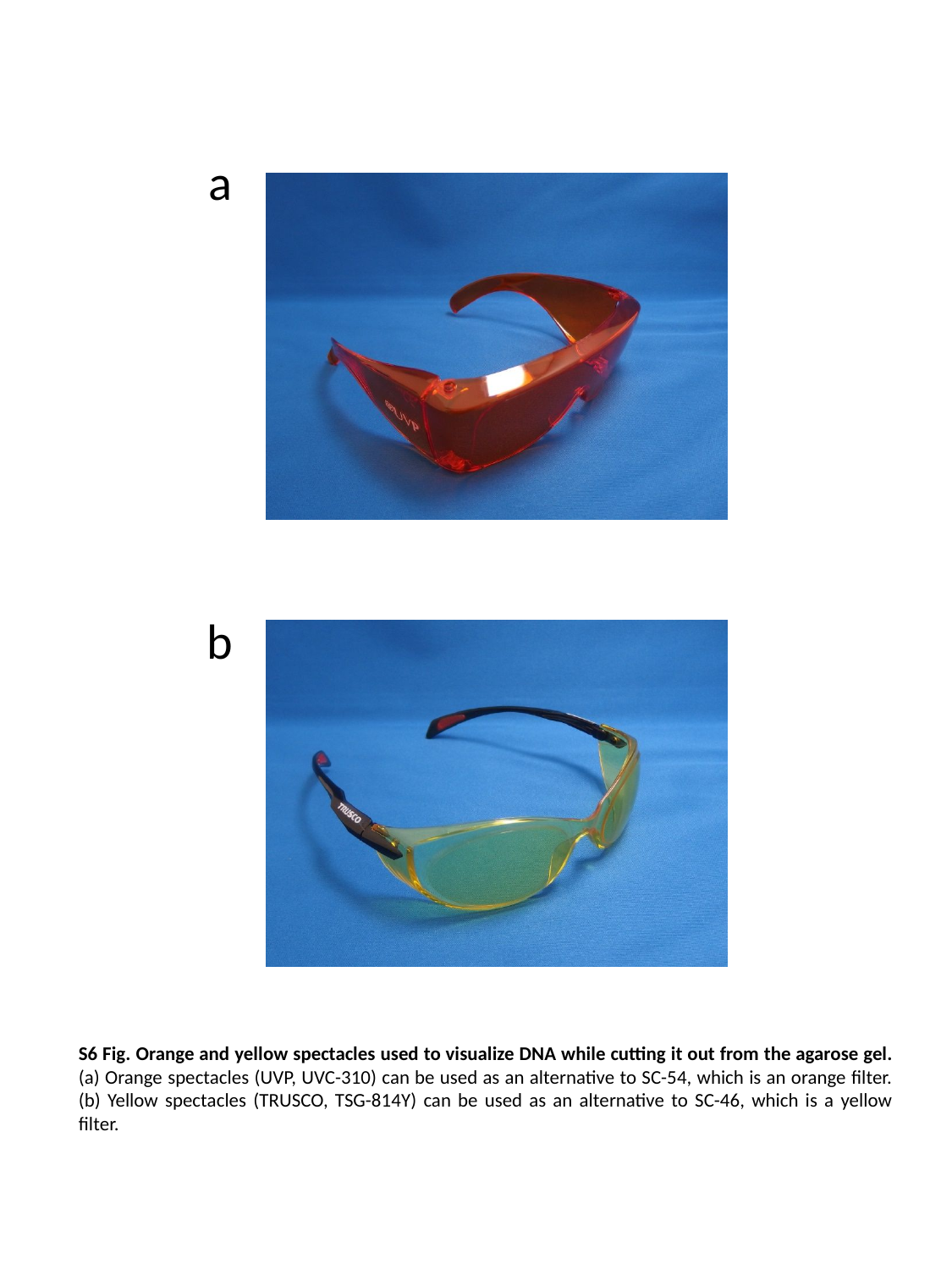

a
b
S6 Fig. Orange and yellow spectacles used to visualize DNA while cutting it out from the agarose gel. (a) Orange spectacles (UVP, UVC-310) can be used as an alternative to SC-54, which is an orange filter. (b) Yellow spectacles (TRUSCO, TSG-814Y) can be used as an alternative to SC-46, which is a yellow filter.
