# Supplementary material for: Development of highly sensitive and low-cost DNA agarose gel electrophoresis detection systems, and evaluation of non-mutagenic and loading dye-type DNA-staining reagents
Source: PLoS One. 2019 Sep 9;14(9):e0222209. doi: 10.1371/journal.pone.0222209 (PMC6733488; doi:10.1371/journal.pone.0222209)
Supplement: S7 Fig — Profiles of detection limit under each condition were analyzed by “Plot Profile” of Image-J. Each excitation system is represented with boxed letters, and DNA-staining reagent is represented by underlined letters. Blue arrows indicate detectable DNA bands. (a) Fig 4A , lane 3; (b) Fig 4B , lane 5. (PPTX) [file pone.0222209.s007.pptx]

## Slide 1
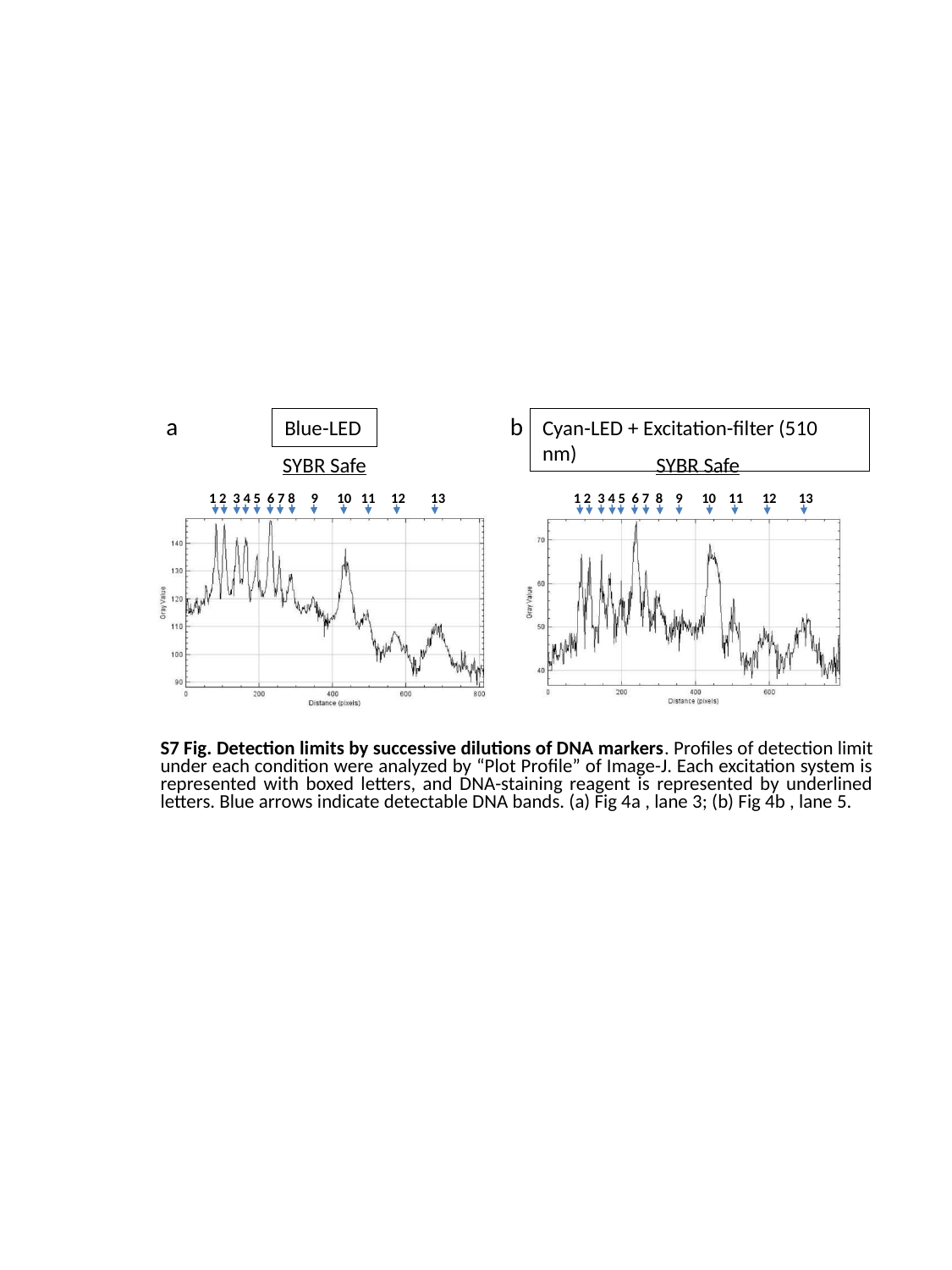

a
b
Cyan-LED + Excitation-filter (510 nm)
Blue-LED
SYBR Safe
SYBR Safe
1 2 3 4 5 6 7 8 9 10 11 12 13
1 2 3 4 5 6 7 8 9 10 11 12 13
S7 Fig. Detection limits by successive dilutions of DNA markers. Profiles of detection limit under each condition were analyzed by “Plot Profile” of Image-J. Each excitation system is represented with boxed letters, and DNA-staining reagent is represented by underlined letters. Blue arrows indicate detectable DNA bands. (a) Fig 4a , lane 3; (b) Fig 4b , lane 5.
